# Increased expression of NOP14 is associated with improved prognosis due to immune regulation in colorectal cancer

**DOI:** 10.1186/s12876-022-02286-x

**Published:** 2022-04-26

**Authors:** Caijie Lu, Weihua Liao, Yiwen Huang, Yaoxing Huang, Yuqi Luo

**Affiliations:** 1 Department of Gastrointestinal and Hepatobiliary Surgery, Shenzhen Longhua District Central Hospital, No. 187, Guanlan Road, Longhua District, Shenzhen, 518110 Guangdong Province China; 2 Department of Radiology, Guangzhou Nansha District Maternal and Child Health Hospital, No. 103, Haibang Road, Nansha District, Guangzhou, 511457 Guangdong Province China; 3 Department of Gastroenterology, Guangzhou First People’s Hospital, School of Medicine, South China University of Technology, Guangzhou, China; 4 Department of Emergency, Nansha Hospital, Guangzhou First People’s Hospital, School of Medicine, Southern China University of Technology, Guangzhou, Guangdong China

**Keywords:** Colorectal cancer, NOP14, Prognostic marker, Immune infiltration, Nucleolar protein

## Abstract

**Background:**

Colorectal cancer (CRC) is the third most common of cancer-related deaths. Nucleolar protein 14 (NOP14) is known to play different roles in diverse types of cancers. However, little is known about its roles in CRC. Here, we assessed the prognostic value and functions of *NOP14* in CRC using the data from The Cancer Genome Atlas (TCGA) and validated them based on the data from Gene Expression Omnibus (GEO).

**Methods:**

NOP14 mRNA and protein data in CRC was obtained from the TCGA, GEO, human protein atlas (HPA), and UALCAN databases. Survival and Cox regression analysis was performed to assess the prognostic value of *NOP14* in CRC patients. Next, to evaluate the potential functions of *NOP14*, a protein–protein interaction (PPI) network was constructed and gene set enrichment analysis (GSEA) of differential expression genes (DEGs) associated with dysregulated *NOP14* was performed. Finally, to investigate the immune response associated with *NOP14* expression in CRC, we analyzed the correlations between immune cells infiltration and *NOP14* expression level. Additionally, the correlations between immune molecule expression levels with *NOP14* expression level were analyzed.

**Results:**

High *NOP14* mRNA expression was observed in CRC tissues based on the data from TCGA and GEO datasets. Similarly, high NOP14 protein levels were found in CRC tissues according to the immunohistochemical images from HPA. Interestingly, high *NOP14* expression level was associated with an improved prognosis in CRC patients. Univariate and multivariate Cox regression analysis indicated that high *NOP14* expression level was an independent protective factor for CRC patients. With the support of PPI network analysis, we found several risk genes interacted with *NOP14*. GSEA revealed that high *NOP14* expression inhibited several signal pathways involved in tumor formation and development. Additionally, high *NOP14* expression was positively associated with most kinds of immune cell infiltrations and the expression levels of some molecules related to immune activation.

**Conclusion:**

Altogether, these results indicated that high *NOP14* expression leads to improved prognosis in CRC patients by inhibiting the signaling pathways involved in tumor growth and promoting the immune responses.

## Introduction

Colorectal cancer (CRC) is a very prevalent cancer worldwide and remains the third leading cause of cancer-related death [1]. The most effective screening test for the early diagnose of CRC is colonoscopy. However, the implementation of colonoscopies in a clinical setting is limited, resulting in approximately 60% of CRC patients being at an advanced stage at the time of diagnosed. Despite the recent progress of surgery, radiotherapy, chemotherapy, and targeted therapy have improved the prognosis of CRC patients, it still remains relatively poor due to the delayed diagnosis. According to the data of China National Cancer Center, the 5-year survival rate of colorectal cancer in China is about 50% [2]. The formation and development of CRC is a complex process associated with increased expression of oncogenes and reduced expression of tumor suppressor genes. Interestingly, *CXCL11*, encoding the chemokine ligand C-X-C motif chemokine ligand 11, has been reported to be significantly upregulated in colon cancer tissues and to be associated with a better prognosis. Additionally, high *CXCL11* expression was reported to be positively correlated with immune infiltration and expression levels of cytotoxic genes, which can activate the antitumor immune response, thereby resulting in improved survival. This exemplifies the importance of studying the role of the genes upregulated in cancer, and as it may contribute to understand the tumorigenic process and reveal potential therapeutic targets [3].

*NOP14* encodes Nucleolar Protein 14, an 875 amino protein, which is highly conserved in eukaryotes and participates in numerous biological processes, such as DNA repair and replication and cell cycle control. A recent study reported *NOP14* regulates the expression of the methyltransferase Emg1, thereby being required for 18 s rRNA maturation [4]. Moreover, increasing studies supported that *NOP14* is involved in cancer initiation and development. For example, NOP14 inhibited the Wnt/β-catenin signaling pathway, which reduced the proliferation and metastasis of melanoma [5] and breast cancer [6], thereby acting as a tumor suppressor gene. Similarly, in bladder cancer, *NOP14* attenuated the miR-502-5p-mediated inhibition of migration and proliferation of tumor cells. Nonetheless, the roles of *NOP14* varies depending on the specific types of cancer. In pancreatic cancer, *NOP14* was highly expressed, and had been shown to promote the growth and invasion of tumor cells in vitro by stabilizing mutant P53, which suppressed the expression of P21 via induction of miR-17-5p [7]. Thereby, *NOP14* acts as an oncogene in some tumors, and as tumor suppressor gene in others.

To date, the role of *NOP14* in CRC has not been reported. In this study, we analyzed the differential expression of *NOP14* between CRC tissues and adjacent normal specimens using the data from the Cancer Genome Atlas (TCGA) and Gene Expression Omnibus (GEO) database, and then evaluated its prognostic value in CRC patients. Next, a protein–protein interaction (PPI) network was construct and Gene Set Enrichment Analysis (GSEA) was performed to predict the potential functions of *NOP14.* Finally, we assessed the correlations between the *NOP14* expression level and immune infiltration as well as immune molecules, to unravel the role of *NOP14* in the prognosis of CRC patients.

## Materials and methods

### Data acquisition, preprocessing, and ethics statement

Pan-cancer RNA sequencing (RNA-seq) data was obtained from UCSC XENA database (https://xenabrowser.net/datapages/). Level 3 RNA RNA-seq expression data and clinical data for CRC (647 CRC tissues vs. 51 normal adjacent cancer tissues) were downloaded from TCGA portal (https://portal.gdc.cancer.gov/). Before analysis, transcripts per million reads (TPM) RNA-seq data was log transformed (Log2(TPM + 1)). ChIP data of expression profiles for CRC samples were downloaded from following GEO datasets (https://www.ncbi.nlm.nih.gov/geo/): GSE 20482 (65 pairs of CRC tumor and matched adjacent non-tumor tissue samples; platform GPL4133), GSE38839 (101 CRC samples and 35 normal tissues, platform GPL10558), GSE87211 (230 CRC samples and 133 normal colorectal mucosa tissues, platform GPL13497), and GSE161158 (250 CRC tissues and supplementary clinical information, platform GPL570). As TCGA and GEO are open publicly available databases, the data collection from the databases was compliant with all applicable laws, regulations, and policies for the protection of human subjects, and all written informed consents were obtained from all subjects involved.

### Analysis of differential expression of *NOP14*

The R package edgeR was used to analyze differentially expressed genes (DEGs) between CRC and normal colon tissue within TCGA database using the threshold parameters |log2(FC)|> 1 and *p* adj value < 0.01. To compare the expression levels of *NOP14* in pan-cancer and CRC, statistical analyses were performed using R (V3.6.3), considering a *p* value of < 0.05 significant. The CRC tissue samples were divided into two groups according to median *NOP14* expression level (high- and low- *NOP14* expression level), among which 3 cases were duplicated, which had been deleted and not included in the analysis. Finally, these samples were used to investigate the prognostic value, and immune infiltration associated with *NOP14* expression levels.

### Analysis of NOP14 protein expression in CRC tissue samples

Immunohistochemistry staining images of NOP14 in CRC and normal tissue sections were downloaded from HPA (https://www.proteinatlas.org/), in which these sections used the same antibodies and experimental methods. Next, the differential expression of NOP14 protein in CRC and normal adjacent tissues was obtained from UALCAN (http://ualcan.path.uab.edu/), which is a web resource including gene transcriptional data and clinical information of cancers from TCGA [8].

### Association of *NOP14* expression with clinical factors in CRC patients

To determine the diagnostic value of *NOP14* expression levels, a receiver operating characteristics (ROC) curve was built using R package pROC (v1.17.0.1), and the area under cure (AUC) with 95% confidence interval (CI) was calculated to confirm the diagnostic efficacy. Next, to assess the potential value of *NOP14* expression on prognostic prediction for pan-cancer and CRC patients, Kaplan–Meier (K—M) survival curves were generated according to *NOP14* expression level and survival status data from TCGA and GSE161158 and analyzed using the R packages survival (v3.2–10) and survminer (v0.4.9). The difference between high- and low- *NOP14* expression groups was analyzed using the log-rank test, using a *p* value < 0.05 as the significance threshold. In addition, 1-, 3- 5-year aspects of time dependent ROC were constructed using the R package timeROC. Similarly, to identify the independent survival risk factors for CRC patients, among gender, age, T and N stages, and *NOP14* expression level, univariate and multivariate Cox regression analysis were performed using the R package survival (v3.2–10). The hazard rate (HR) with 95% confidence interval (CI) and *p* value were presented with forest plots using R package ggplot2 (v3.3.3).Next, a nomogram and a calibration curve were constructed based on the results of univariate and multivariate Cox regression analysis of *NOP14* expression level and clinical feature using the rms package.

### PPI network construction

To explore the proteins interacted with NOP14, a PPI network was constructed using STRING 11.5 ((www.string-db.org) [9]. The interacting proteins were selected based on the original criteria of STRING 11.5. The linkage scores were used to identify the interaction pairs.

### Functional enrichment analysis of DEGs

GSEA associated with DEGs between the high- and low- *NOP14* expression level groups was performed using the clusterProfiler package, with c2.cp.v7.2.symbols.gmtcurated gene sets from MSigDB Collections as reference gene sets [10, 11]. Clusters with a *p* value < 0.05 and a false discovery rate (FDR) < 0.25 were considered significant.

### Immune infiltration analysis

Single-sample GSEA (ssGSEA) was used to assess the infiltration of individual immune cell populations, e.g., T helper 2 cells (Th2 cells), CD8 T cells, NK CD56dim cell, and others. The analysis was performed using GSVA package [12]. Next, the correlations between NOP14 expression level and tumor purity, B cell, CD8 + cell, CD4 + cell, macrophage, neutrophil and dendritic cell infiltration were calculated and plotted using TIMER2.0 (http://timer.cistrome.org/) [13, 14].

### Analysis of immune-related molecules levels

To investigate the association between the expression levels of *NOP14* with immune-related molecules, a Pearson’s correlation analysis was performed using a *p*-value < 0.05 as the threshold for significance. Heat maps of the significant immune-related molecules based on Pearson coefficients were generated using the R package ggplot2.

### Statistical Analyses

Statistical analyses were performed using R (v.3.6.3). The ggplot2 package was used for data visualization. Data are presented as means ± standard deviation (SD). All tests were two-sided, and a *p* < 0.05 was defined as statistically significant.

## Results

### *NOP14* expression in pan-cancer and CRC patients

**Fig. 1:**
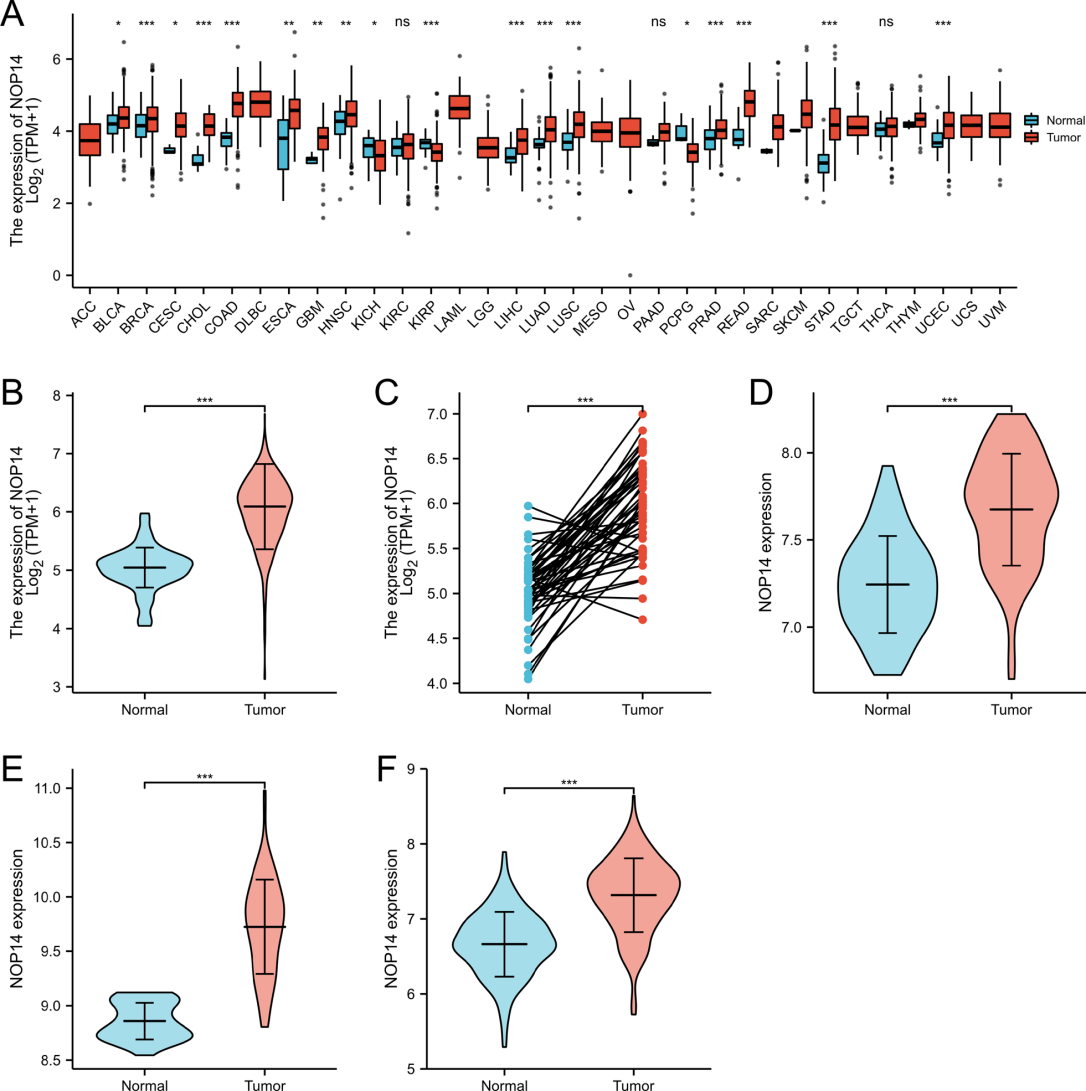
*NOP14* expression pattern in pan-cancer and colorectal cancer (CRC) based on data from The Cancer Genome Atlas (TCGA) and Gene Expression Omnibus (GEO). **A** *NOP14* was highly expressed in 18 of 33 cancers compared with its expression in normal tissues. ***p < 0.001, **p < 0.01, *p < 0.05; n.s., no significance. **B** *NOP14* expression is significantly higher in tumor tissues than in normal tissues. ***p < 0.01. **C** *NOP14* expression in 51 CRC tissues and their paired adjacent para-carcinomatous tissues. *** *p* < 0.001. **D–F** Differential *NOP14* expression between cancer and normal tissues according to the datasets GSE 20,482 (**D**), GSE38839 (**E**), and GSE87211 (**F**). ***p < 0.01

**Fig. 2:**
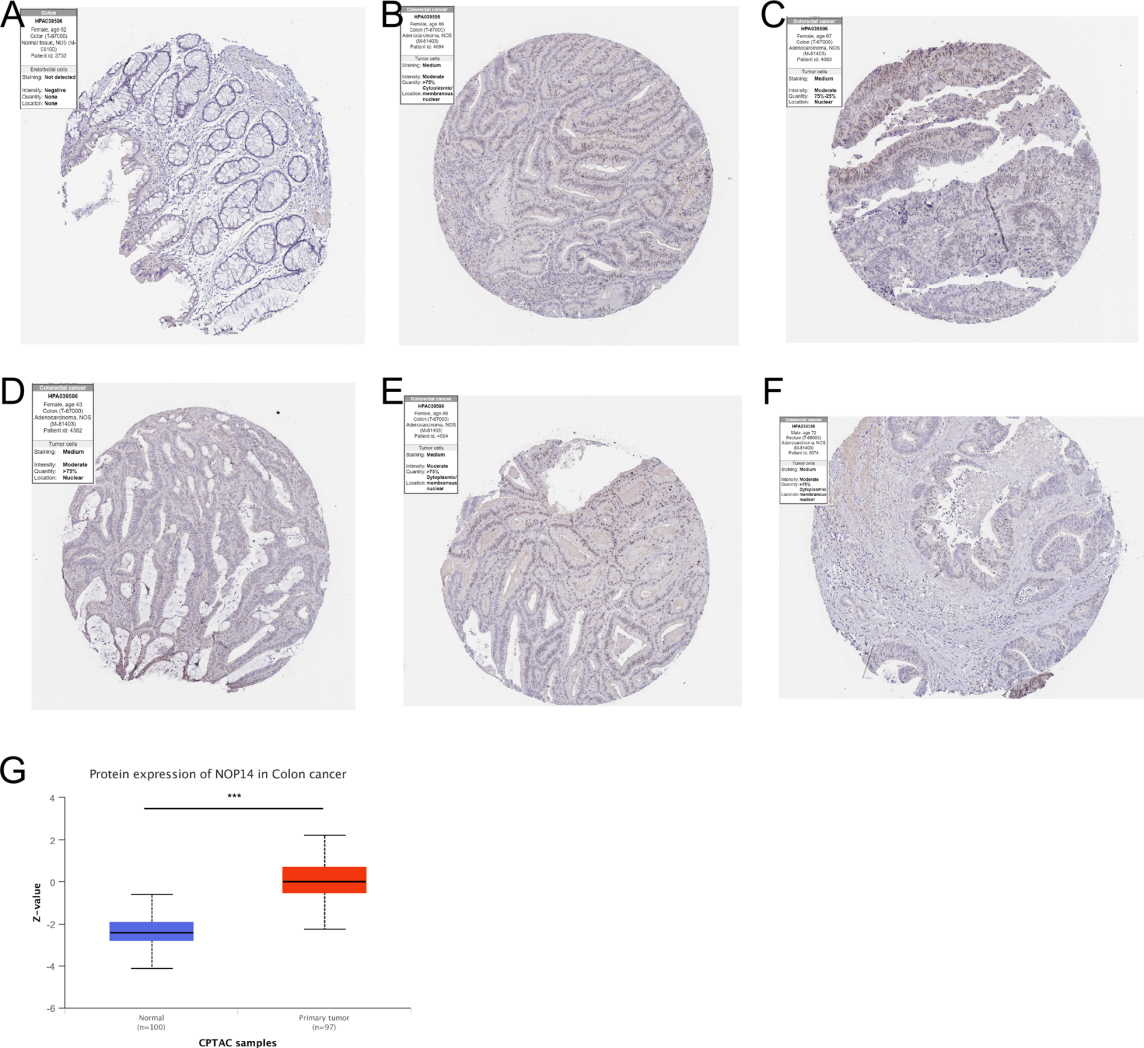
NOP14 protein expression pattern in colorectal cancer (CRC) tissues. NOP14 protein was not detected in normal colon tissues (**A**), and was detected in CRC tissues with moderate signal intensity (**B–F**) based on the immunohistochemical images from Human Protein Atlas (HPA). (**G**) NOP14 protein expression level was significantly higher in CRC tissues than in normal tissues based on the data from UALCAN, ***p < 0.01

The clinical features of the 644 CRC patients included in this study are shown in Table 1. Using the data from TCGA, we found that *NOP14* expression was significantly elevated in 19 of 33 cancer types, including bladder urothelial carcinoma (BLCA), colon adenocarcinoma (COAD), cholangiocarcinoma (CHOL), liver hepatocellular carcinoma (LIHC), lung squamous carcinoma (LUSC), stomach adenocarcinoma (STAD), among others (Fig. 1A). Similarly, *NOP14* expression was significantly increased in CRC tissues compared with normal samples (Fig. 1B). For the 51 pared samples, *NOP14* expression was also significantly higher in cancer tissues than in matched normal adjacent tissue samples (Fig. 1C). These results were confirmed in three GEO datasets: GSE 20482, GSE38839, and GSE87211 (Fig. 1D–F). Next, NOP14 protein expression levels were assessed using immunohistochemistry images obtained from HPA. Whereas NOP14 protein was not detected in normal tissue (Fig. 2A), but NOP14 protein positive cells were moderate in tumor tissues (Fig. 2B–F). Similarly, NOP14 protein expression level was significantly higher in cancer tissues than in normal tissue samples according to the data from UALCAN (Fig. 2G).

**Table 1:** Relationship between NOP14 expression levels and clinical features of colorectal cancer patients based on data from TCGA

Characteristic	Low *NOP14* expression	High *NOP14* expression	*p*
n	322	322	
Gender, n (%)			0.527
Female	155 (24.1%)	146 (22.7%)	
Male	167 (25.9%)	176 (27.3%)	
Age, n (%)			0.032
≤ 65	124 (19.3%)	152 (23.6%)	
> 65	198 (30.7%)	170 (26.4%)	
T stage, n (%)			0.890
T1	10 (1.6%)	10 (1.6%)	
T2	55 (8.6%)	56 (8.7%)	
T3	214 (33.4%)	222 (34.6%)	
T4	40 (6.2%)	34 (5.3%)	
N stage, n (%)			0.209
N0	176 (27.5%)	192 (30%)	
N1	76 (11.9%)	77 (12%)	
N2	68 (10.6%)	51 (8%)	
M stage, n (%)			0.395
M0	235 (41.7%)	240 (42.6%)	
M1	49 (8.7%)	40 (7.1%)

### Clinical significance associated with *NOP14* expression in CRC patients

**Fig. 3:**
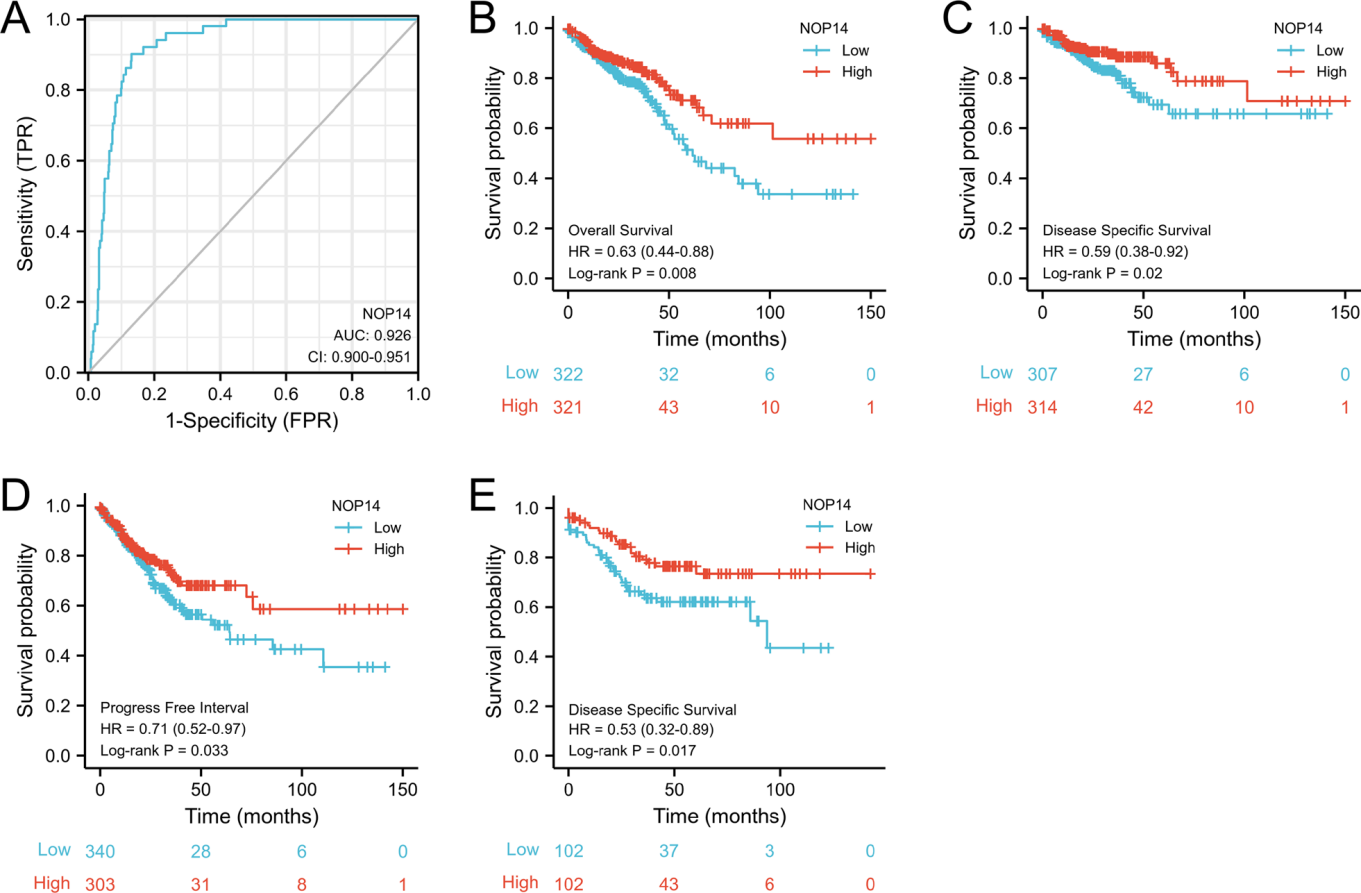
*NOP14* expression level has potential diagnostic and prognostic values for patients with colorectal cancer (CRC). **A** Receiver operating characteristic (ROC) curve for NOP14 (area under the curve [AUC]: 0.926, *P* < 0.001). Kaplan–Meier (K–M) survival curves for overall survival (OS) (**B**), disease specific survival (DSS) (**C**), and disease free interval (DFI) (**D**) constructed based on data from The Cancer Genome Atlas (TCGA). **E** K–M survival curves for DSS constructed based on data from the Gene Expression Omnibus (GEO) dataset GSE161158

To evaluate the diagnostic value of NOP14 expression level, a ROC curve was constructed. As shown in Fig. 3A, the *NOP14* expression had a high sensitivity and specificity for CRC diagnosis. The area under the curve (AUC) was 0.926, and Youden index was 0.771. Next, the prognostic value of *NOP14* expression was evaluated using a K–M analysis. According to the data from TCGA, the overall survival (OS), disease specific survival (DSS), and progress free interval (PFI) were significantly better in high *NOP14*-expression group than in low *NOP14*-expression group (Fig. 3B–D). Additionally, we validated the DSS results in the cohort from the GEO dataset GSE161158 (HR:0.53, *p* < 0.05, Fig. 3E). Interestingly, as shown in Table 2, high *NOP14* expression levels were associated with different prognostic impacts in different types of cancers. For example, in colon adenocarcinoma (COAD), stomach adenocarcinoma (STAD), rectum adenocarcinoma (READ), and kidney renal clear cell carcinoma (KIRC), high *NOP14* expression levels were associated with a good prognosis (HR < 1, *p* < 0.05). Whereas in brain lower grade glioma (LGG), liver hepatocellular carcinoma (LIHC), sarcoma (SARC), adrenocortical carcinoma (ACC), high expression levels of *NOP14* were associated with a poor prognosis (HR > 1, *p* < 0.05).

### Prognostic efficacy of *NOP14* expression levels

**Fig. 4:**
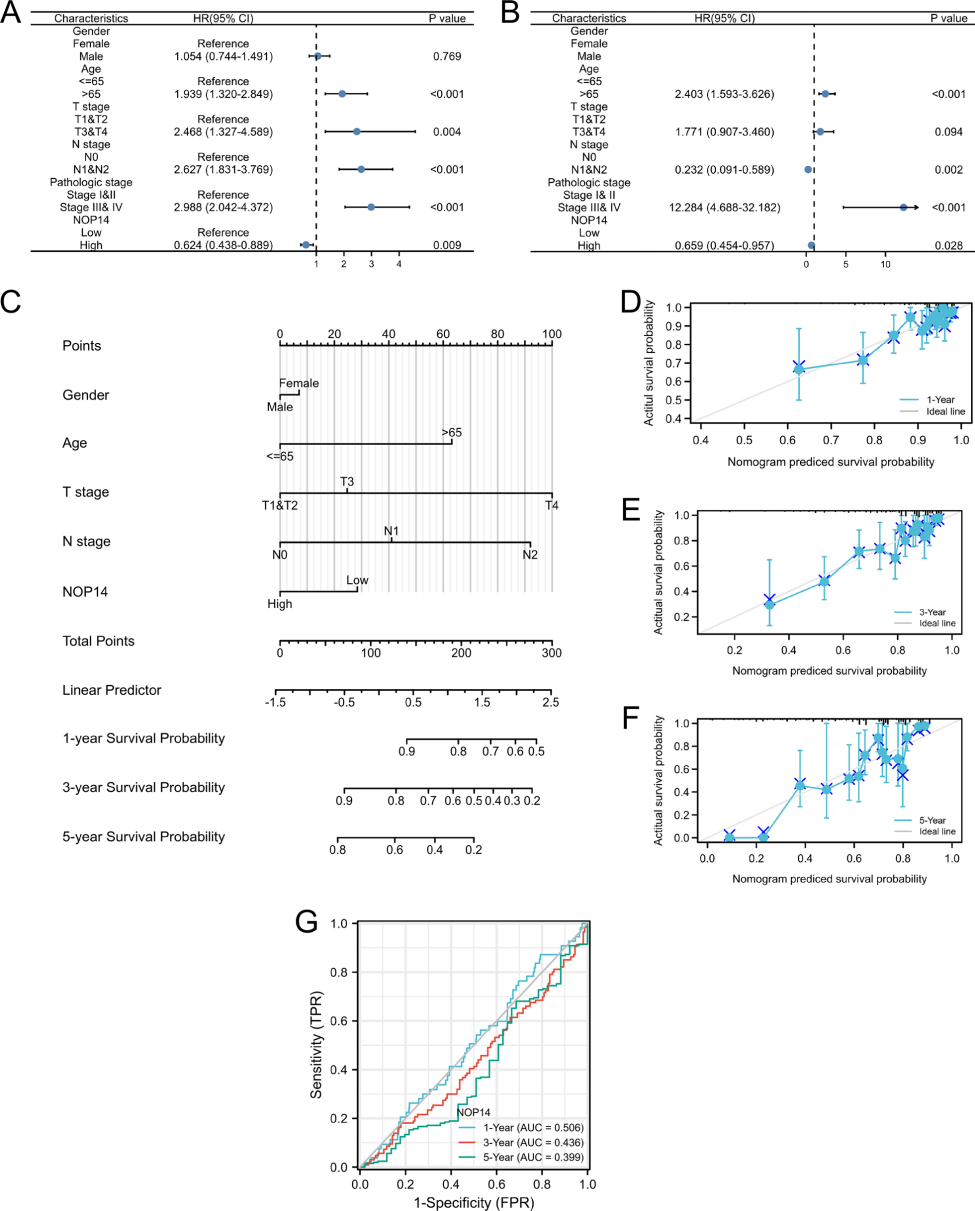
Prognostic efficacy of *NOP14* expression levels in patients with colorectal cancer (CRC). Results of univariate (**A**) and multivariate (**B**) Cox regression analyses displayed using forest plots. **C** Nomogram for predicting clinical outcomes associated with *NOP14* expression in CRC patients. **D–F** Calibration plots validating 1-, 3-, and 5-year clinical outcomes for CRC patients. **G** Time-dependent receiver operating characteristic (ROC) analysis of 1-, 3-, and 5-year overall survival based on *NOP14* expression levels in CRC patients

We then performed univariate and multivariate Cox regression analyses using several clinical features and *NOP14* expression level. As shown in Fig. 4A, B, high *NOP14* expression was an independent protective factor for OS in CRC patients. In contrast, advanced age (> 65 years old) and clinical stage (T3-4 stage and N1 stage) predicted poor clinical outcomes. Then, we generated a simple-to-use nomogram based on clinical features, including gender, age, T and N stage, as well as *NOP14* expression level. As shown in Fig. 4C, *NOP14* expression level had a good performance in predicting 1-, 3- and 5-years survival rate in the patients with CRC. The concordance index (C-index) was 0.726 (95% CI: 0.701–0.751). Similarly, the calibration plots ran very close to the diagonals, which showed a good performance in calibrating (Fig. 4D–F). Time dependent ROC revealed similar results for 1-, 3- 5-year follow-up, the AUCs were < 0.5, indicating that high *NOP14* expression might be a protective factor for CRC patients (Fig. 4G).

### Proteins interacting with NOP14

**Fig. 5:**
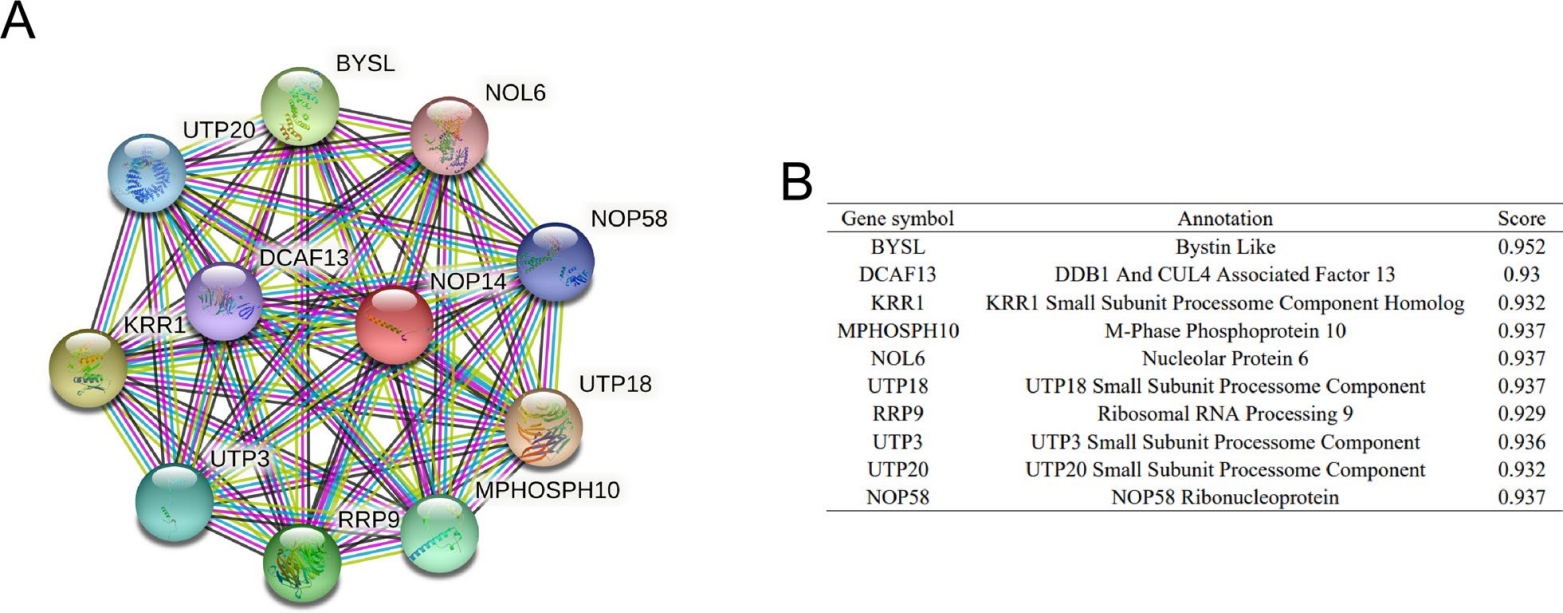
Proteins interacting with NOP14. **A** Protein–protein interaction (PPI) network of NOP14. **B** Annotation of proteins that interact with NOP14 and their co-expression scores

To explore the proteins interacted with NOP14, we generated a PPI network of NOP14 protein using the tool STRING (Fig. 5A). The top 10 proteins and their gene symbol, annotation, and scores are listed in Fig. 5B. The proteins interacted with NOP14 include BYSL, DCAF13, KRR1, MPHOSPH10, NOL6, UPT20, RRP9, UPT3, UPT18, and NOP58, some of which closely related to carcinogenesis.

### Functional enrichment analysis of the genes associated with *NOP14* expression level 

**Fig. 6:**
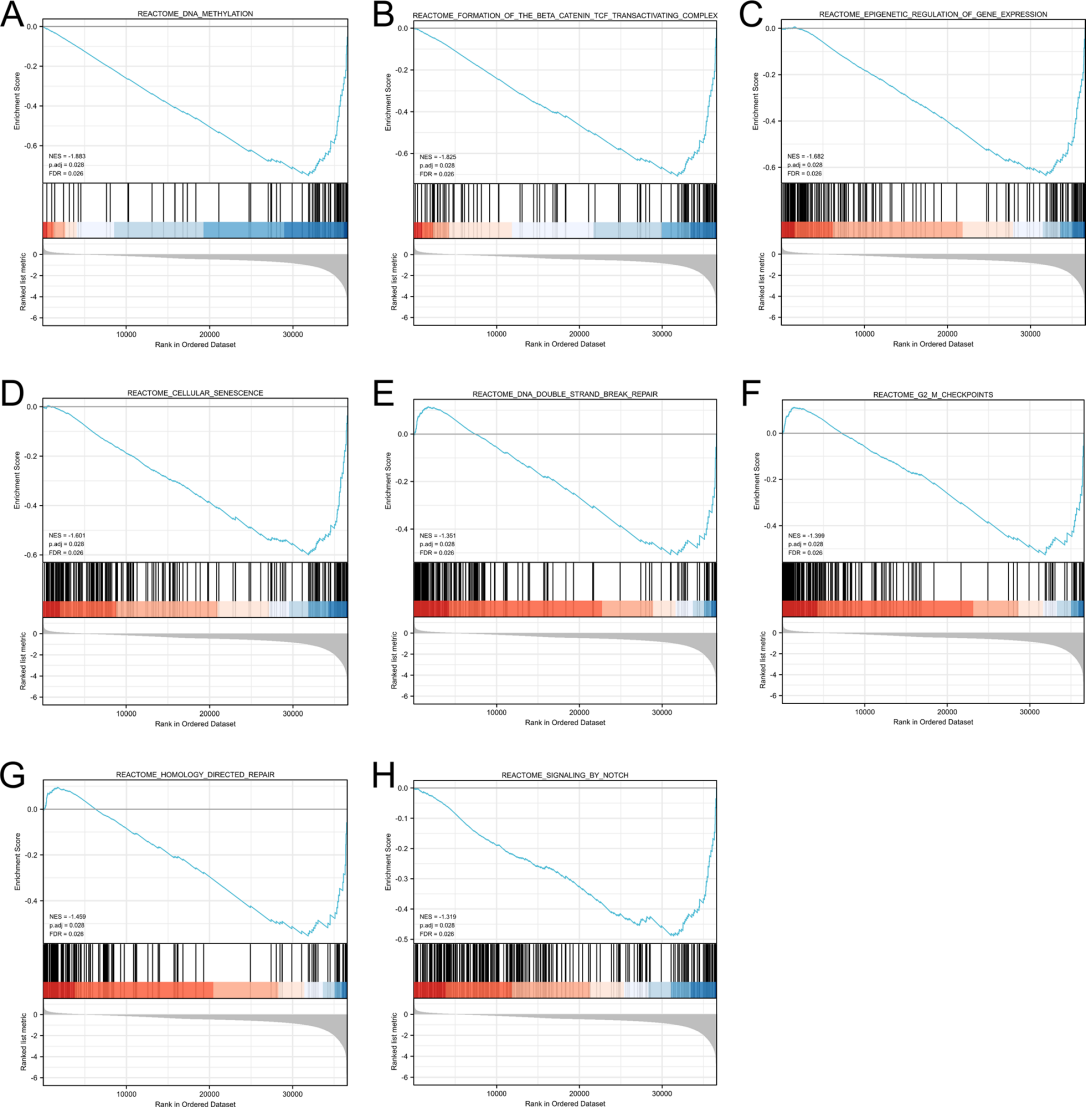
Biological pathways enriched in NOP14 low-expression group (A–H) determined using Gene Set Enrichment Analysis (GSEA). NES, normalized enrichment score

To further investigate the potential function of NOP14 in CRC, CRC samples were divided into *NOP*14 low- and high- expression groups, and DEGs were identify, and GSEA was performed. The results of GSEA revealed that some pathways associated with tumor cell growth were significantly enriched in *NOP14* low- expression group (*p* < 0.05, FDR < 0.025), e.g., FORMATION_OF_THE_BETA_CATENIN_TCF_TRANSACTIVATING_COMPLEX, DNA_METHYLATION, SIGNALING_BY_NOTCH (Fig. 6A-H).

**Fig. 7:**
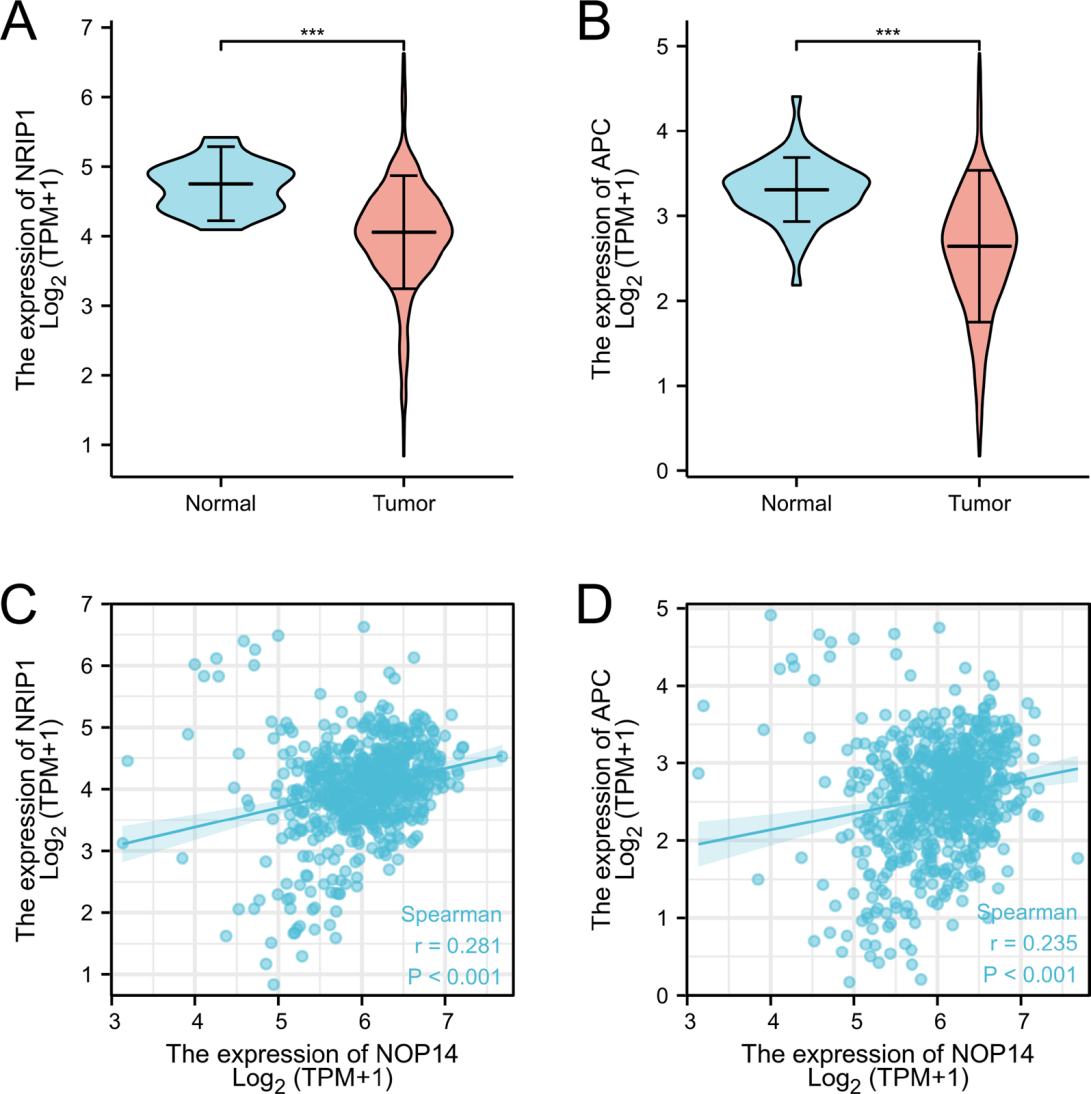
*NOP14* expression pattern associated with inhibitors of the Wnt/catenin pathway. NRIP1 (**A**) and APC (**B**) expression is significantly lower in cancer tissues than in normal tissues. *** *p* < 0.001. Positive correlations between NOP14 and NRIP1 (**C**) and NOP14 and APC (**D**) expression are shown. *p* < 0.001

Next, we analyzed the expression levels of the inhibitors of the Wnt/β-catenin signaling pathway, Nuclear Receptor Interacting Protein 1 (NRIP1) and Adenomatous Polyposis Coli (APC) in CRC tissues in CRC tissues. The expression levels of NRIP1 and APC were significantly lower in tumor tissues than normal samples (Fig. 7A, B). Additionally, *NOP14* expression levels were positive correlated to NRIP1 and APC expression levels (*p* < 0.01, Fig. 7C, D). Therefore, *NOP14* might act as a tumor suppressor gene through inhibiting some tumor growth-related signal pathways in CRC cells, such as Wnt/β-catenin pathway.

### Association between *NOP14* expression and immune cell infiltration and immune molecule expression levels

**Fig. 8:**
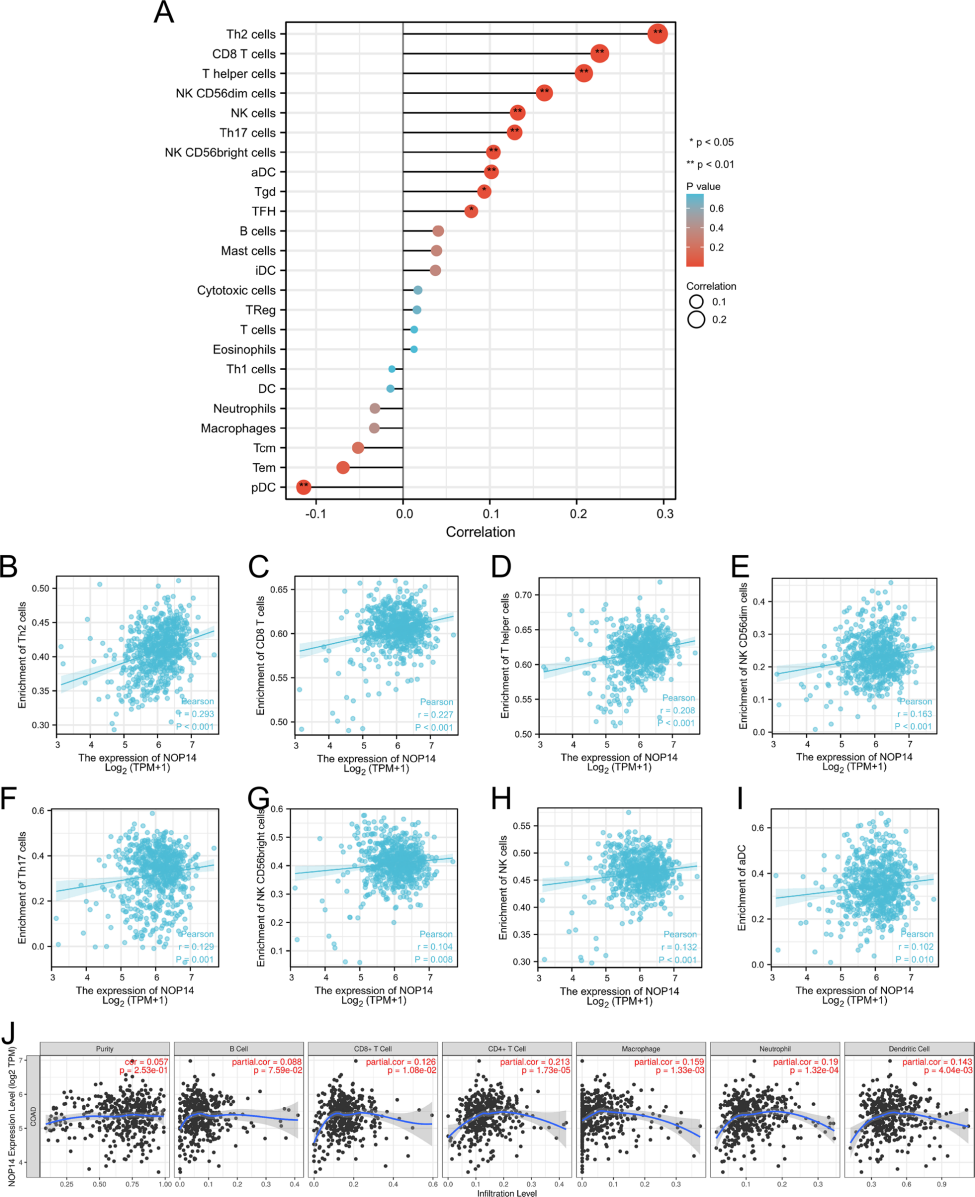
Relationship between *NOP14* expression and immune cell infiltration in the tumor microenvironment. **A** Correlations between *NOP14* expression and 24 types of immune cell infiltrations. **B–I** Correlations between T helper (h) 2, cluster of differentiation (CD)8 + , Th, NK CD56^dim^, Th17, NK CD56^bright^, NK, and B cell and aDC enrichment levels and *NOP14* expression. **J** Correlations between *NOP14* expression and infiltrations of CD8+, CD4+, B, and dendritic cells as well as macrophages and neutrophils determined using TIMER2.0 tool

**Fig. 9:**
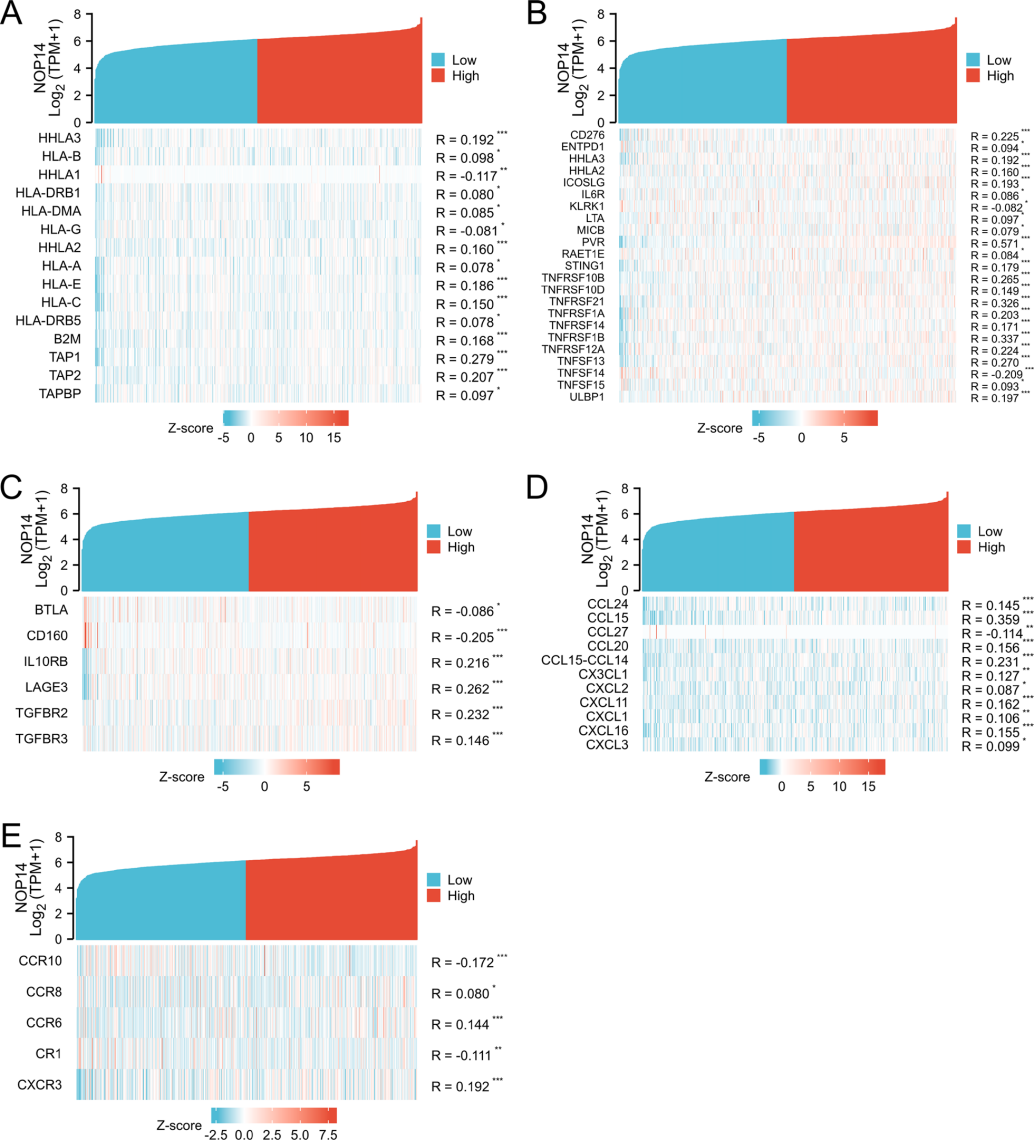
Coexpression heatmaps for correlations between expression of *NOP14* and immune-related factors. The heatmaps display the expression levels of MHC genes, immune activation genes (**A**), immunosuppressive genes (**B**), chemokine receptors (**C**), and chemokines (**D**) significant correlated with *NOP14* expression

To understand the effects of NOP14 expression on tumor microenvironment, immune infiltration analysis was performed using ssGSEA method. The correlations between enrichments of immune cell in CRC tissues and NOP14 expression levels were calculated using Spearman correlation analyses. As shown in Fig. 8A–I, *NOP14* expression levels were positively correlated with numerous types of immune cells, e.g., Th2 cells, CD8 T cells, T helper cells, NKCD56 dim cells, NK cells, and aDC cells, all of which play an important role in anti-tumor immunity. Next, to observe the infiltrations of other immune cells in CRC tissues, TIMER2.0 was used to analyze the correlations between *NOP14* expression levels and the infiltrations of CD8 + cells, CD4 + cells, B cells, macrophages, neutrophils, and dendritic cells. As shown in Fig. 8J, *NOP14* expression levels were significantly positively correlated with the infiltrations of those immune cells (*p* < 0.01). Similarly, the correlations between *NOP14* expression levels and the expression levels of several immune-related molecules, such as MHC genes (Fig. 9A), immune activation genes (Fig. 9B), immunosuppressive genes (Fig. 9C), chemokine receptors (Fig. 9D) and chemokines (Fig. 9E) were also statistically significant.

## Discussion

CRC is a prevalent malignant tumor of the digestive tract. Despite the prognosis of CRC has remarkably improved in the last years due to the progress in the fields of diagnosis and therapy, the prognosis of advanced CRC patients remains unsatisfactory. Therefore, it is urgent to find new molecular markers to improve the ability of predicting the prognosis of CRC. In this study, we assessed the genes that highly expressed in tumor tissues and was associated with a good prognosis in CRC patients. According the data from TCGA and GEO, we found that *NOP14* expression was significantly higher than normal samples. Interestingly, high *NOP14* expression was associated with improved prognosis for CRC patients, and acted as an independent protective factor. The underlying mechanism might be related to the inhibition of signaling pathways associated with tumor growth, and the activation of immune cell infiltration and immune response.

*NOP14*, which is an evolutionally conserved protein in eukaryotes, mainly locates in nucleus and nucleus membrane and plays an important role in pre-18 s rRNA processing and small ribosomal subunit assembly [15]. It has been recently reported that *NOP14* can be considered as an immune regulator factor, and that is involved in various diseases [16, 17]. In chronic inflammatory disease, *NOP14* activates NF-κB signaling pathway, and upregulates proinflammatory gene expression, and increases the expression of adhesion molecules in endothelial cells, thereby promoting the infiltrations of immune cells [18]. This highlights that *NOP14* is closely related with immune response and that it plays an important role in biological processes of various cells.

The involvement of *NOP14* in cancer initiation and development is still unclear. Some studies have reported that *NOP14* expression levels are associated with prognosis of various types of cancer [19–21]. In our study, we showed that *NOP14* expression was significantly elevated in many kinds of cancer, such as bladder urothelial carcinoma, cholangiocarcinoma, and liver hepatocellular carcinoma. Because colon adenocarcinoma and rectum adenocarcinoma share similar biological features, we integrated them as CRC during the analysis processes. DEGs analysis showed that *NOP14* expression level was also significantly higher in CRC tissues than in normal tissues, suggesting in may play an important role in carcinogenesis and development of CRC, among other cancers. So far, only a few studies have reported the prognostic value of *NOP14* expression levels in cancer. Isaksson et al. reported that low expression of *NOP14* associated with poor prognosis in ovarian cancer [21]. Similarly, Chang et al. found that *NOP14* highly expressed in colon cancer tissues, and that high expression predicted a good prognosis [20].We further investigated this relationship, and found that the high expression of *NOP14* had different effects on OS for different cancers (Table 2). In COAD, STAD, READ, and KIRC, high *NOP14* expression level was associated with an improved prognosis. However, in LGG, LIHC, SARC, and ACC, high *NOP14* expression level was associated with a poor prognosis. These results suggest that *NOP*14 plays different roles in different cancers. For CRC patients, according to the results of K–M survival analyses based the data from TCGA and GEO, we found that high *NOP14* expression level was associated with an improved prognosis. Furthermore, Univariate and multivariate Cox regression analysis identified that high *NOP14* expression level as an independent protective factor for CRC patients. Our nomogram and calibration plots confirmed that *NOP14* expression level was a good predictor for 1-, 3, 5-year OS in CRC patients. Therefore, *NOP14* had a good performance in prognostic prediction, illustrating that *NOP14* could be used as a biomarker of diagnosis and therapeutic target.

**Table 2:** NOP14 expression levels significantly related to overall survival of patients with 8 eight types of cancer according to the data from TCGA

Cancer types	HR	95% CI	*P* value
STAD	0.69	0.50–0.96	0.028
READ	0.41	0.19–0.89	0.029
COAD	0.59	0.40–0.88	0.015
KIRC	0.62	0.45–0.84	0.002
LGG	1.75	1.25–2.45	0.001
LIHC	1.49	1.06–2.11	0.021
SARC	1.59	1.07–2.37	0.02
ACC	2.24	1.06–4.73	0.035

Stomach adenocarcinoma (STAD), rectum adenocarcinoma (READ), colon adenocarcinoma (COAD), kidney renal clear cell carcinoma (KIRC), lower grade glioma (LGG), liver hepatocellular carcinoma (LIHC), sarcoma (SARC), adrenocortical carcinoma (ACC)

Based on previous literature, we hypothesized that the effects mediated by *NOP14* in tumor formation and metastasis were mediated by the Wnt/β-catenin pathway. In melanoma and breast cancer, *NOP14* inhibited the proliferation and metastasis of tumor cells by down regulating the Wnt/β-catenin signal pathway [5, 6]. In this study, we found that Wnt/β-catenin signal pathway was enriched in *NOP14* low expression group, indicating that high *NOP14* expression might downregulate the pathway, acting as a tumor suppressor gene. Nonetheless, some other pathways associated with tumor growth were also enriched in the low *NOP14* expression group, e.g., Notch signaling, DNA methylation, G2/M checkpoints. Next, we analyzed the expression levels of the key proteins in Wnt/β-catenin pathway. NRIP1, a transcription co-regulatory factor, negatively regulated the Wnt/β-catenin pathway by increasing APC expression level and degrading β-catenin [22, 23]. Consistent with previous studies, we found that NRIP1 and APC expression were significantly lower in CRC tissues than in normal tissues. However, their expression levels were significantly positively correlated with *NOP14* expression levels, suggesting that NOP14 might upregulate NRIP1 and APC expression in CRC tissues. To further unravel the biological function of NOP14 in CRC, we generated a PPI network using the tool STRING. As shown in Fig. 5, we found that NOP14 interacted with some proteins closely related to tumor initiation and development, such as BYSL or NOL6. Mechanistically, BYSL activated the AKT pathway by regulating RIOK2 and mTOR, acting as an oncogene in gliomas [24]. NOL6 promoted the proliferation and migration of various kinds of tumor cells, including endometrial cancer, hepatocellular carcinoma, and prostate cancer [25–27]. Therefore, the biological functions of NOP14 are closely related to carcinogenesis and development of CRC.

Many reports supported that immune cell infiltration in tumor tissues associate with tumor formation, growth, and metastasis [28, 29]. A high concentration of immune-promoting cells in tumor tissue improved patient prognosis, whereas a low concentration of immune-promoting cells may lead to immune escape of cancer cells, resulting in a poor prognosis [30, 31]. In this study, we found that *NOP14* expression levels were positively correlated to the infiltration levels of numerous immune cells, such as CD8 cells, CD4 cells, NK cells, and dendritic cells, suggesting that *NOP14* might promote the anti-tumor immune response in tumor tissues. Additionally, *NOP14* expression levels were significantly correlated with the expression levels of many immune molecules, like MHC genes, immune activation genes, chemokine receptor, and chemokines, which might recruit immune cell infiltration, thereby exerting an anti-tumor effect. Altogether, these results suggest that *NOP14* may exert an anti- oncogenic function by triggering anti-tumor immune responses in CRC tissues.

**Fig. 10:**
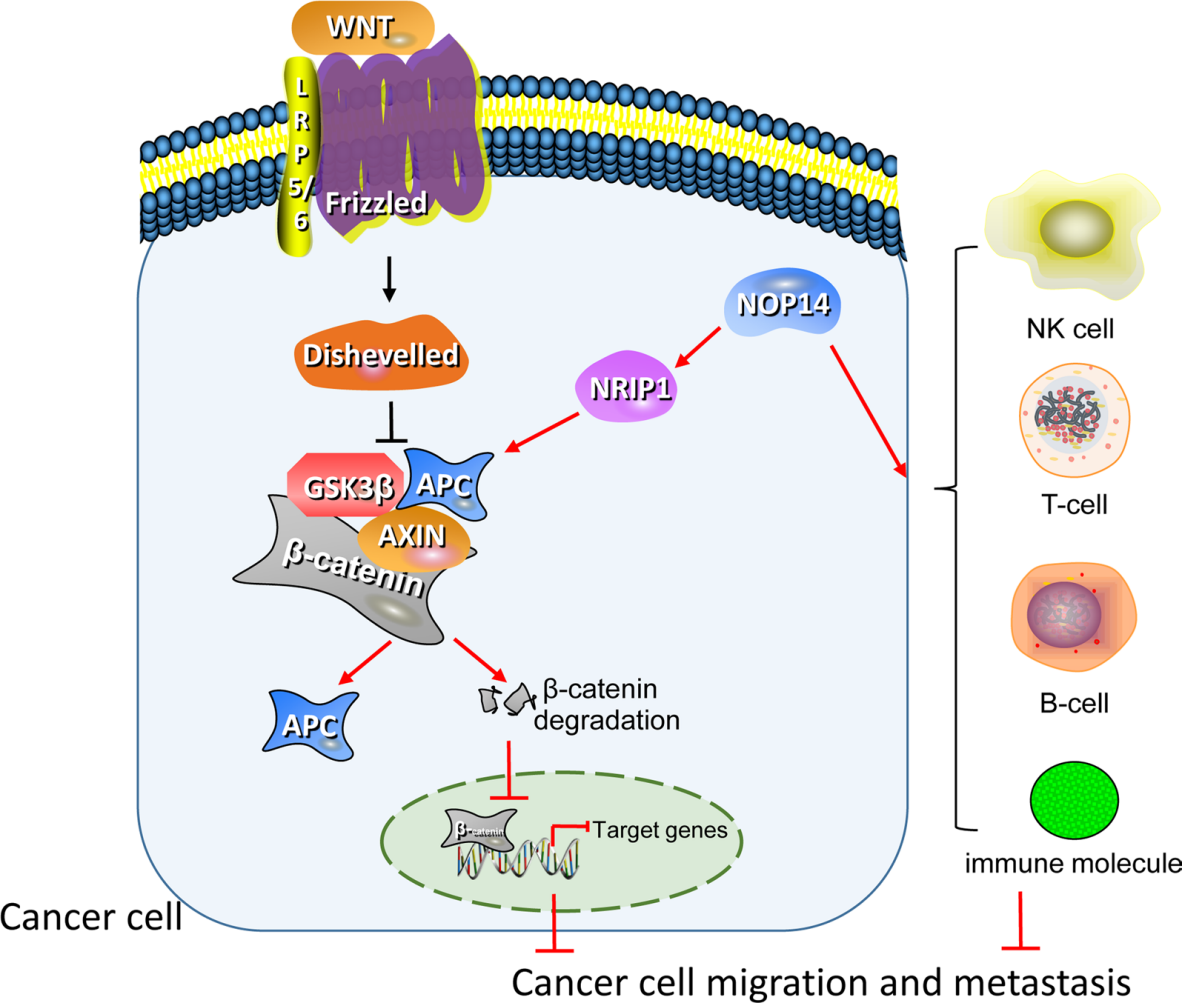
Schematic diagram of a possible mechanism of action of NOP14 in colorectal cancer (CRC) formation and metastasis, that is inhibiting the Wnt/β-catenin pathway and promoting anti-tumor response

In conclusion, we preliminarily conformed that *NOP14* is as a prognostic biomarker in CRC patients. Additionally, we suggested the potential mechanism relying on inhibiting the pathways of tumor growth and promoting anti-tumor immune response (Fig. 10). Nonetheless, further research is necessary to confirm this mechanism of action.

## Data Availability

The data used for analysis in this study was obtained from public available databases, which were UCSC XENA (https://xenabrowser.net/datapages/), TCGA data portal (https://portal.gdc.cancer.gov/), and GEO database (https://www.ncbi.nlm.nih.gov/geo/).
